# The subtle nuances of intranasal corticosteroids

**DOI:** 10.1186/s40463-020-00480-z

**Published:** 2021-03-17

**Authors:** James Fowler, Brian W. Rotenberg, Leigh J. Sowerby

**Affiliations:** Department of Otolaryngology – Head and Neck Surgery, Western University, 3St. Joseph’s Hospital, 268 Grosvenor Street, London, ON N6A 4V2 Canada

**Keywords:** Intranasal corticosteroid, Rhinitis, Rhinosinusitis

## Abstract

**Background:**

In the specialty of Otolaryngology – Head and Neck Surgery, intranasal corticosteroids are the mainstay treatment for inflammatory processes within the nasal cavity. All too often, physician prescribing patterns are based on previous training, personal experience, and interactions with industry. The purpose of this commentary is to review the nuances of each intranasal corticosteroid.

**Commentary:**

There are nine intranasal corticosteroids approved for use in Canada. Each are discussed in detail, including their indication, bioavailability, effects on intranasal environment, and factors around patient adherence. Off-label use of budesonide irrigations is also discussed and cost information is presented in reference format for all available intranasal corticosteroids.

**Conclusion:**

Although the efficacy of each intranasal corticosteroid has been shown to be similar, prescribing should be tailored based on bioavailability, intranasal environment, and factors that impact patient adherence such as dosing, cost and tolerability.

**Graphical abstract:**

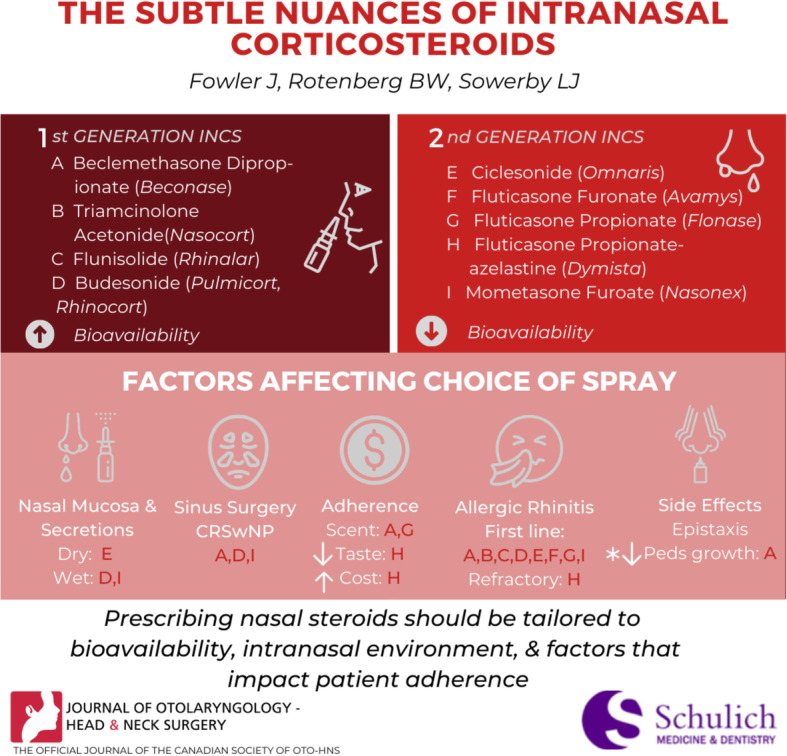

## Background

In the specialty of Otolaryngology – Head and Neck Surgery, intranasal corticosteroids (INCS) s are the mainstay treatment for inflammatory processes within the nasal cavity. Currently, there are nine INCSs available in Canada (Table 1). The utility of INCSs can be appreciated by their pharmacodynamics. Structurally, the steroids are different, but they all work in a similar manner. Intranasal corticosteroids affect both early and late inflammatory response by inhibiting the production of pro-inflammatory cytokines, inflammatory enzymes, lymphocyte proliferation, and delayed hypersensitivity [4].

**Table 1:** Summary of intranasal corticosteroids available in Canada, including indication, bioavailability, fragrance, and cost

Intranasal Corticosteroid	Indications [1, 2]	Bioavailability [1, 3]	Fragrance	Cost per Bottle Brand (Generic)	Cost per Spray Brand (Generic)
**Beclomethasone dipropionate**^**@**^ Beconase AQ (50 mcg)	• Allergic rhinitis • Non-allergic rhinitis • Chronic rhinosinusitis with NP	**44%**	Scented	$31 ($14)	16¢ (7¢)
**Budesonide**^**@**^ Rhinocort Aqua (64 mcg,^1^ 100 mcg^2^) Pulmicort nebule (0.25^3^,0.5^4^, 1 mg^5^) Rhinocort Turbuhaler* (100 mcg^*6*^*-off market)*	• Allergic rhinitis • Non-allergic rhinitis • Chronic rhinosinusitis with nasal polyposis • Wet nasal congestion (Turbuhaler)	Spray: **31%** Turbuhaler: **22%**	Non-scented	1. $13 ($12) 2. $26 ($22) 3. $11(N/A)* 4. $23 (N/A)* 5. $42 ($32)* 6. $29 (N/A) *per box of 20	1. 11¢ (10¢) 2. 16¢ (13¢) 3. 54¢ (43¢)* 4. $1.15 (N/A)* 5. $2.10 ($1.60)* 6. 15¢ (N/A) *cost per nebule
**Ciclesonide** Omnaris (50 mcg)^@^	• Perennial allergic rhinitis • Seasonal allergic rhinitis • Dry nasal congestion	< 1%	Non-scented	$32 ($N/A)	27¢ (N/A)
**Flunisolide** Rhinalar (25 mcg)	• Seasonal allergic rhinitis	**49%**	Non-scented	N/A (N/A)	N/A (N/A)
**Fluticasone – azelastine** Dymista (137 mcg/50 mcg)	• Seasonal allergic rhinitis	0.8%	Non-scented	$120 (N/A)	$1 (N/A)
**Fluticasone furoate** Avamys (27.5 mcg)	• Allergic rhinitis • Non-allergic rhinitis	0.5%	Non-scented	$33 (N/A)	27¢ (N/A)
**Fluticasone propionate** Flonase (50 mcg)	• Allergic rhinitis • Non-allergic rhinitis	0.5%	Scented	$36 ($25)	30¢ (20¢)
**Mometasone furoate**^**@**^ Nasonex (50 mcg)	• Allergic rhinitis • Acute sinusitis • Chronic rhinosinusitis with nasal polyposis • Adenoid hypertrophy • Wet nasal congestion	0.5%	non-scented (was previously scented)	$36 ($24)	30¢ (20¢)
**Triamcinolone acetonide**^**@**^ Nasacort AQ (55 mcg)	• Allergic rhinitis	**46%**	Non-scented	$24 ($22)	20¢ (19¢)

Cost data from October 2020 based on averaged information from two Ontario pharmacies. Does not include dispensing fee. ^@^ indicates coverage by Ontario Drug Benefit.

Intranasal corticosteroids are the primary monotherapy, or adjunct therapy, for many rhinological conditions. Numerous studies have shown their effectiveness in treating allergic/non-allergic rhinitis, [2] acute rhinosinusitis, [3] chronic rhinosinusitis with nasal polyposis (CRSwNP) [2], chronic rhinosinusitis without polyposis (CRSsNP [2], and adenoid hypertrophy [5]. Objectively, INCSs have demonstrated significant efficacy for reducing relative/instantaneous total nasal symptom scores (rTNSS and iTNSS, scored out of 24) [6–10], relative/instantaneous total occular symptom scores (rTOSS and iTOSS, scored out of 18) [6, 10–12], and endoscopic polyposis scores (scale 0–3) [13–18]. Regular use of INCSs is also associated with increases in peak nasal inspiratory flow [9, 19, 20] and improved quality of life (Rhinoconjunctivitis Quality of Life Questionnaire) [6, 8, 9, 20].

Although the efficacy for each INCS is roughly equivalent [4], there are subtle characteristics to be considered when prescribing for a patient. All too often, physician prescribing patterns are based on previous training, personal experience, and interactions with industry. The purpose of this paper is to review the nuances of each INCS, including their indication, bioavailability, intranasal environment, and patient adherence.

## Review

### Selection of intranasal corticosteroid

Intranasal corticosteroids can be categorized based on their generation. The older, first generation INCSs (beclomethasone dipropionate, triamcinolone acetonide, flunisolide, budesonide) have a significantly higher systemic bioavailability than the second generation INCSs (ciclesonide, fluticasone furoate, fluticasone propionate, mometasone furoate) (Table 1).

Characteristics of the nasal mucosa can also alter selection of INCS. The presence, or absence, of nasal secretions affects how the INCS is absorbed. Movement of nasal cilia can become impaired if the sol layer of mucous is too thin, or too thick [21]. This affects the permeability of the steroid, as mucociliary clearance is altered, and duration of contact between the steroid and nasal mucosa is decreased [21]. To counteract this, selection of an INCS that alters the viscosity within the nose, can increase contact time and overall diffusion of the steroid. For example, in a “dry” congested nose, ciclesonide is favoured. Ciclesonide is a hypotonic solution, resulting in rapid diffusion water molecules into the nasal mucosa of a dry nose. The difference in osmolarity increases the viscosity within the nose, thus increasing contact time [22]. A similar principle can be seen for a “wet” congested nose. In this case, mometasone furoate and budesonide (Rhinocort Turbuhaler) are recommended. Mometasone furoate contains the highest concentration of microcrystalline cellulose and carboxymethylcellulose sodium for aqueous INCS [23]. These are thixotropic agents, which dry and increase viscosity within the nasal cavity. Rhinocort Turbuhaler also dries the nose, secondary to its dry powered formulation, but as of February 2020 has been discontinued by the manufacturer. Both steroids increase viscosity, while decreasing moisture within the nasal cavity.

**Fig. 1:**
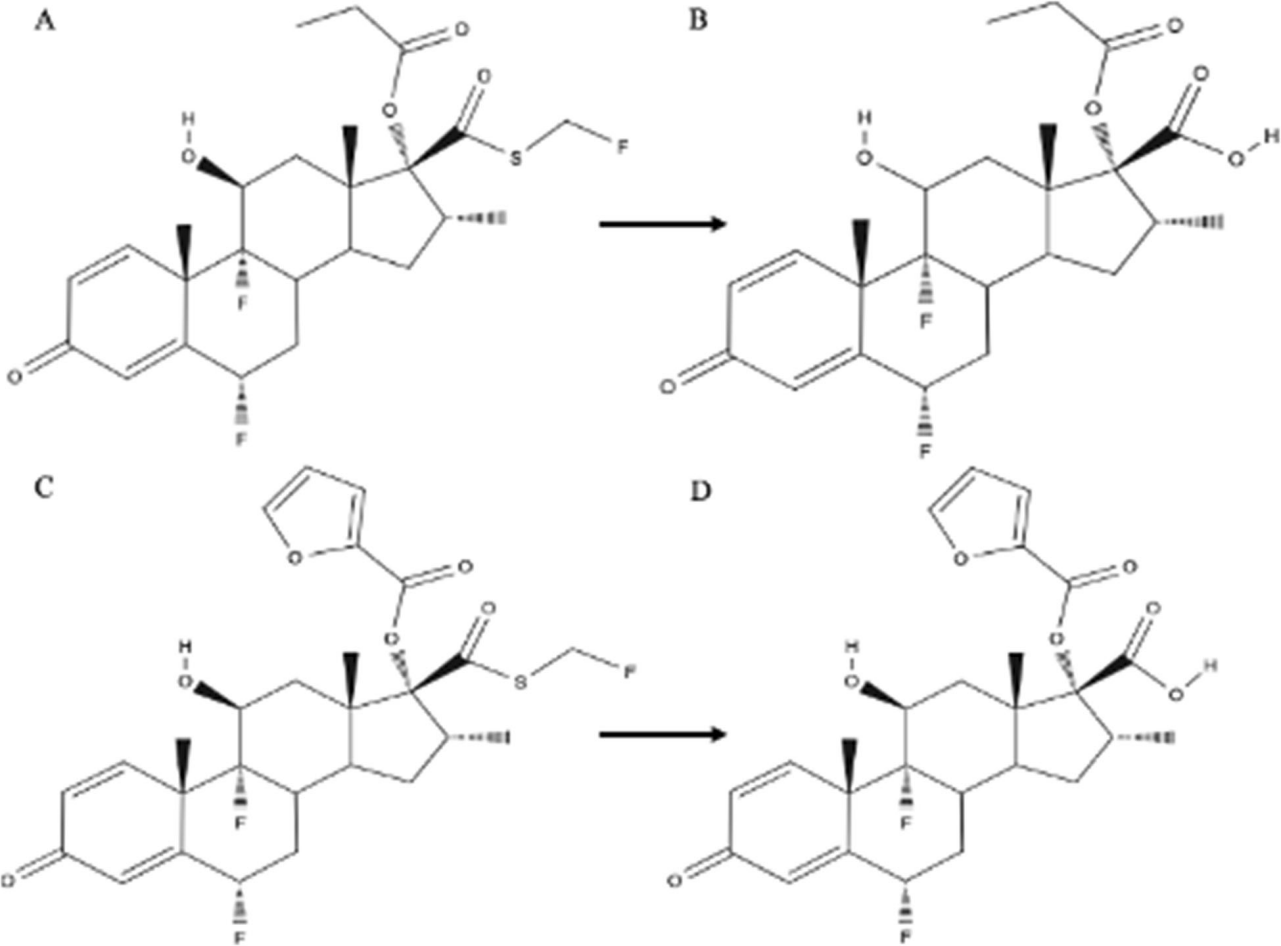
Metabolism of fluticasone propionate (**a**) and fluticasone furoate (**c**) to their 17-carboxylic acid metabolites (**b** and **d**, respectively)

Although fluticasone is the backbone of both fluticasone furoate (FF - Avamys) and fluticasone propionate (FP - Flonase), one should be cognisant that their efficacies are not equivalent. The two molecules are relatively similar in structure, only differing in the esters attached to the 17α-OH group (Fig. 1a-d). Esterified furoate and propionic acid are found at this location for fluticasone furoate and fluticasone propionate, respectively [24]. When metabolized, fluticasone is not released from the ester substituent, which affects target receptor binding [25]. The ester sidechain of fluticasone furoate is much larger than that of fluticasone propionate. This structural difference allows fluticasone furoate to bind to the glucocorticoid receptor with a higher affinity [26]. Valotis et al. [27] report that fluticasone furoate has a relative receptor affinity ratio (in comparison to dexamethasone) of 2989, while fluticasone propionate has an affinity of 1775. Clinically, this results in superior efficacy for fluticasone furoate (Avamys) [24, 26, 28].

Fluticasone propionate-azelastine (Dymista) is unique. Produced in 2012, it combines the therapeutic effects of a corticosteroid and an antihistamine. Fluticasone-azelastine is effective in treating severe seasonal allergic rhinitis refractory to steroid or antihistamine treatment alone [1]. The combination spray results in an incremental improvement of around 1 over and above FP on the Total Nasal Symptom Score (TNSS), and doubles the small reduction seen on the Total Ocular Symptom Score (TOSS) with FP alone [29]. Dymista achieved a reduction of TNSS to one or less in 12% of patients, versus 9.5% for FP, and had 35% of patients achieve a 50% reduction in TNSS by day 7, versus day 9 for INCS alone and day 11 for azelastine [29]. The bioavailability of the fluticasone component is low, but is 44–61% higher than monotherapy FP [30]. This is a negligible difference as the bioavailability of monotherapy FP is < 0.5% [1]. Adverse effects are rare (< 5%), and have a similar side effect profile to other INCSs [1]. Given the cost-benefit ratio, it generally should be reserved as a second line therapy after failure of another INCS.

Budesonide nebules (Pulmicort) are approved as a maintenance therapy for asthma. In recent years, this medication has been used “off-label” to treat CRSwNP before or after endoscopic sinus surgery and has become widely accepted as maximal medical therapy for this condition. Budesonide nebules are produced in various concentrations (0.25 mg, 0.5 mg, and 1 mg per 2 mL) and are mixed with saline to irrigate the nasal cavity. Multiple studies have shown that budesonide irrigations are efficacious in treating nasal polyposis by improving both Lund-Kennedy endoscopic scores and Sinonasal Outcomes Test (SNOT-22) scores [31–33]. The dosage used in reported trials is between 128 μg-2 mg per day, divided per nostril either once or twice daily [2]. The exact bioavailability of the budesonide irrigation is unknown, but it is believed to be less than typical INCSs, as less than 5% of the solution remains in the sinuses when administered with a squeeze bottle [34]. Systemic side effects are minimal, as very little solution remains in the nasal cavity. In two short-term safety studies, no evidence of HPA axis suppression was found [35, 36]. In regards to long-term safety, Smith et al. evaluated patients using 2 mg of budesonide in daily irrigations for an average of 3 years without evidence of any hypothalamic-pituitary-adrenal (HPA) axis suppression in 35 patients [37]. Soudry et al. [38] evaluated 48 patients after a mean duration of 22 months with daily budesonide irrigations (mean daily dose of 0.75 mg) and all demonstrated normal intraocular pressures. They did, however, find low levels of stimulated cortisol in 11 (23%) patients but all were without symptoms of adrenal suppression. Risk of suppression was strongly associated with concurrent pulmonary steroid inhalers and suppression reversed in 75% after cessation of budesonide rinses for 1 month [38]. As such, for patients on concurrent inhaled corticosteroids consideration should be given to using the lowest budesonide dose that can control symptoms. A referral to endocrinology to test for subclinical HPA axis suppression via ACTH stimulation testing is an option in select cases, but is not currently part of routine standard practice.

Lastly, symptom management is dependent on adherence to the INCS. Three factors affecting INCS adherence are patient preference, ease of use, and affordability. Patient preference is strongly related to sensory attributes associated with INCS. Scent and aftertaste of the INCS greatly impact regular use. As sensory attributes increase, patient preference for the INCS decreases [39, 40]. Scented INCS, (beclomethasone dipropionate, fluticasone propionate) often have an accompanied aftertaste. Fluticasone-azelastine also has a strong aftertaste. If a patient has reported sensitivity to scents, it is recommended to prescribe a non-scented formulation. Use of a mouthwash gargle prior to application of the spray can help mitigate this side-effect. Delivery is also important to consider – some of the bottles have longer nozzles that are easier to place in the nasal cavity, whereas Avamys has a side-action to activate, increasing ease of use with issues of manual dexterity. With respect to affordability, many generic INCS are covered by provincial drug plans. If the INCS is not listed on the designated formulary, it is important to have a discussion with the patient about additional drug coverage, or willingness to pay out-of-pocket. Coverage by the Ontario Drug Benefit and the estimated costs per spray based on data from Ontario in October 2020 are included in Table 1.

### Side-effects

In general, the risk of systemic side effects for INCSs are very low [41]. An early study by Skoner et al. [3] showed that beclomethasone dipropionate had a negative impact on pediatric growth velocity. In this study, 90 children with perennial rhinitis were randomly assigned to a treatment or placebo group. The treatment group received beclomethasone dipropionate 168 μg twice daily for 1 year. Patient height was measured monthly with a stadiometer. At the end of the study, the mean change in height was 5 cm and 5.9 cm, for the treatment and placebo respectively. The authors noted that slowing of the growth was evident within the first month of beclomethasone dipropionate use. Mometasone furoate [42], fluticasone propionate [43], triamcinolone acetonide [44], fluticasone furoate [45], and ciclesonide [46] have all since been studied for growth impairment in pediatric patients, and none of these INCSs impaired growth. With respect to HPA axis suppression, a review by Sheth [47] revealed no correlation between HPA axis suppression and traditional INCSs. Of note, long term off-label use of budesonide for treatment of CRSwNP has been correlated in one study with subclinical HPA axis suppression in patients on concurrent inhaled corticosteroids [27]. It is important to note that this suppression was without clinical manifestations of adrenal sufficiency, and all patients were able to continue corticosteroid therapy safely, and at least three other studies have found no evidence of HPA axis suppression [35, 36]. Lastly, a recent systematic review by Valenzuela et al. [48] analysed the effect of INCSs on intraocular pressure. Their meta-analysis found that there was no association with increased intraocular pressure and INCSs. There were also no diagnoses of glaucoma at 12 months of regular use. While these studies are reassuring that the amount of corticosteroid absorbed is quite low, there is a theoretical increased risk for patients on concurrent oral/inhaled corticosteroids. In this setting, a second generation INCS, with low bioavailability, would be recommended.

Local effects, such as burning/stinging (< 10%), dryness (< 10%), and epistaxis (< 10%), are common regardless of bioavailability [4]. The incidence of epistaxis is higher with some sprays, such as mometasone (12.7%), fluticasone propionate (19%), and fluticasone furoate (20%) with prolonged use [49]. Septal perforation is reported as a complication, but the incidence is very low (< 0.001%), and clear causality has not been established [50].

## Conclusion

In summary, INCSs have become the mainstay conservative management for many rhinological conditions. Within the literature, studies have shown the efficacy of INCSs to be very similar. Although this statement is true, there are subtle nuances that should be considered when selecting medication to prescribe. Prescribing should be tailored based on bioavailability, intranasal environment, and factors that impact patient adherence.

## Data Availability

Not applicable.

## Abbreviations

CRSsNP: Chronic rhinosinusitis without nasal polyposis
CRSwNP: Chronic rhinosinusitis with nasal polyposis
FF: Fluticasone furoate
FP: Fluticasone propionate
HPA: Hypothalamic-pituitary-adrenal
INCS: Intranasal corticosteroid
iTNSS: Instantaneous total nasal symptom score
iTOSS: Instantaneous total occular symptom score
rTNSS: Relative total nasal symptom score
rTOSS: Relative total occular symptom score
SNOT-22: Sinonasal Outcomes Test
TNSS: Total nasal symptom score
TOSS: Total occular symptom score
